# Supplementary material for: Disentangling the impact of Atlantic Niño on sea-air CO2 flux
Source: Nat Commun. 2023 Jun 20;14:3649. doi: 10.1038/s41467-023-38718-9 (PMC10282012; doi:10.1038/s41467-023-38718-9)
Supplement: Supplementary file 1 — Supplementary Information [file 41467_2023_38718_MOESM1_ESM.pdf]

Supplementary Information

Here, we would like to provide the supplementary information for Disentangling the impact of Atlantic Niño on sea-air CO_2 fluxes. Supplementary Information consists of 2 table, 12 figures and reference list cited by Supplementary Information.

CMIP6 Model Name	$RMSE_{SST}$	$RMSE_{CO2}$	*r* of SST	*r* of CO_2	$^{*}RMSE_{CO2\_\Delta}$	*r* of CO_{2_Δ}
CESM2[36]	1.704	0.3480	<u>0.625</u>	0.391	0.175	-0.571
CESM2-WACCM[37]	1.847	0.4256	0.555	0.283	0.199	-0.249
CMCC-ESM2[38]	**1.091**	0.3425	**0.836**	*0.482*	0.175	<u>0.443</u>
CNRM-ESM2-1[39]	1.682	1.057	-0.016	0.224	0.374	-0.427
GFDL-CM4[40]	1.654	0.7791	0.479	0.286	0.139	*0.481*
GFDL-ESM4[41]	1.653	**0.3105**	0.007	0.343	0.177	0.196
IPSL-CM6A-LR[42]	**0.675**	0.4479	*0.833*	<u>0.472</u>	**0.102**	0.434
MIROC-ES2L[43]	1.947	**0.2674**	-0.032	**0.626**	0.189	0.228
MPI-ESM1-2-HR[44]	1.363	0.3918	0.397	0.273	**0.114**	0.384
MPI-ESM1-2-LR[45]	2.040	0.8181	-0.349	-0.336	0.176	0.323
MRI-ESM2-0[46]	**1.084**	<u>0.3362</u>	**0.894**	**0.500**	**0.121**	**0.684**
UKESM1-0-LL[47]	*1.210*	*0.3213*	0.496	0.279	*0.129*	**0.582**
NorESM2-LM	<u>1.274</u>	**0.2664**	**0.873**	**0.585**	<u>0.136</u>	**0.562**

Table S1.

CMIP6 statistical performance.

CMIP6 models (1) assessed in this study and their respective root mean squared error (RMSE) of June-July climatological sea surface temperature (SST) and sea-air CO_2 flux and spatial correlation (*r*) of SST and sea-air CO_2 flux with respect to observation within 6°S-2°N and 45°E-0° (including the SST cold tongue and outgassing maximun of CO_2 flux) box for column 1 to 4. The RMSE and r of composite CO_2 flux anomalies during Atlantic Niño (estimated over 6S-5N and 45W-10E, including the whole di-pole pattern of CO_2 flux anomalies in the observation) are given in columns 5 and 6. Note that the RMSE of CO_2 anomlaies are scaled by composite anomalies of Atlantic-3 index. Bold font indicates the three best model values, italic and underscore font depict 4th and 5th best models.

Name of calculartion \ Input data	SSS	SST	ALK	DIC	S-norm ALK	S-norm DIC	Silicate	Phosphate
pCO_{2F}	Monthly	Monthly	Monthly	Monthly	Not used	Not used	Climatology	Climatology
pCO_{2S}	Monthly	Climatology	Climatology	Climatology	Not used	Not used	Climatology	Climatology
pCO_{2T}	Climatology	Monthly	Climatology	Climatology	Not used	Not used	Climatology	Climatology
pCO_{2A}	Climatology	Climatology	Monthly	Climatology	Not used	Not used	Climatology	Climatology
pCO_{2D}	Climatology	Climatology	Climatology	Monthly	Not used	Not used	Climatology	Climatology
pCO_{2AD}	Climatology	Climatology	Monthly	Monthly	Not used	Not used	Climatology	Climatology
pCO_{2A_S}	Climatology	Climatology	Not used	Climatology	Monthly	Not used	Climatology	Climatology
pCO_{2D_S}	Climatology	Climatology	Climatology	Not used	Not used	Montly	Climatology	Climatology

Table S2. List of *p*CO2 decomposition

List of pCO_2 decomposition estimated from NorESM2 and the corresponding input variables used in the CO2SYS module (2) . Name of each pCO_2 calculation with the CO2SYS module applying different combination of input data of the NorESM2 simulation. Except for sea surface salinity (SSS) and sea surface temperature (SST), the data at the first layer of the ocean component is utilized for the calculation. “S-norm” denotes the variable normalized by salinity (see the Method section for more details).

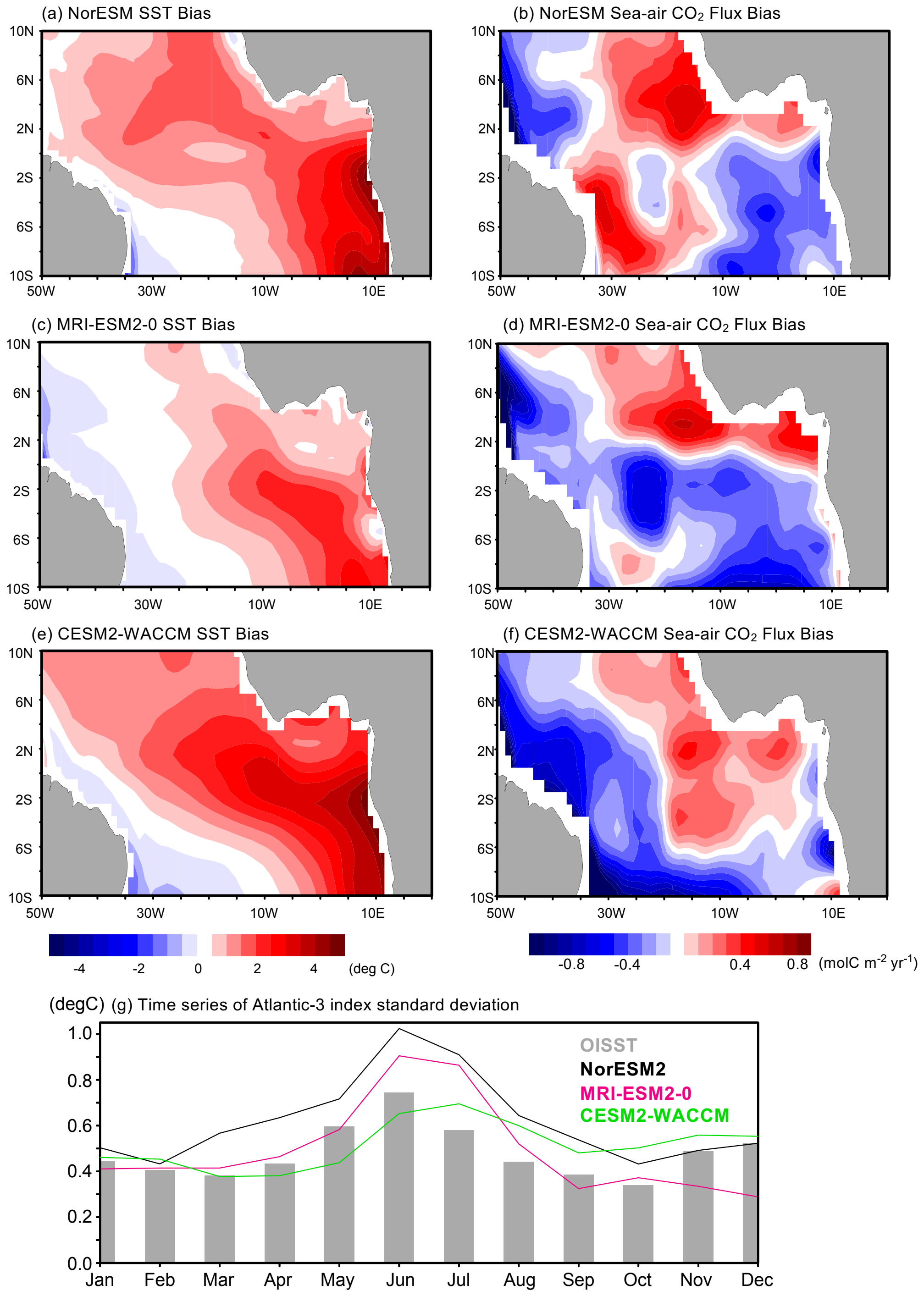

Figure S1. Brief evaluation of NorESM2 and subset of CMIP6 models with respect to observations.

June-July climatology bias of (a), (c), and (e) sea surface temperature and (b), (d), and (f) sea-air CO_2 flux for NorESM, MRI-ESM2-0, and CESM2-WACCM, respectively. Observed SST and CO_2 flux are obtained from 1990-2015 (3) and 1990-2015 (4, 5), respectively. (bottom) time series of Atlantic-3 index standard deviation for observation (gray), NorESM2 (black), MRI-ESM2-0 (magenta), and CESM2-WACCM (green), respectively. Note that one ensemble member is used for all the models.

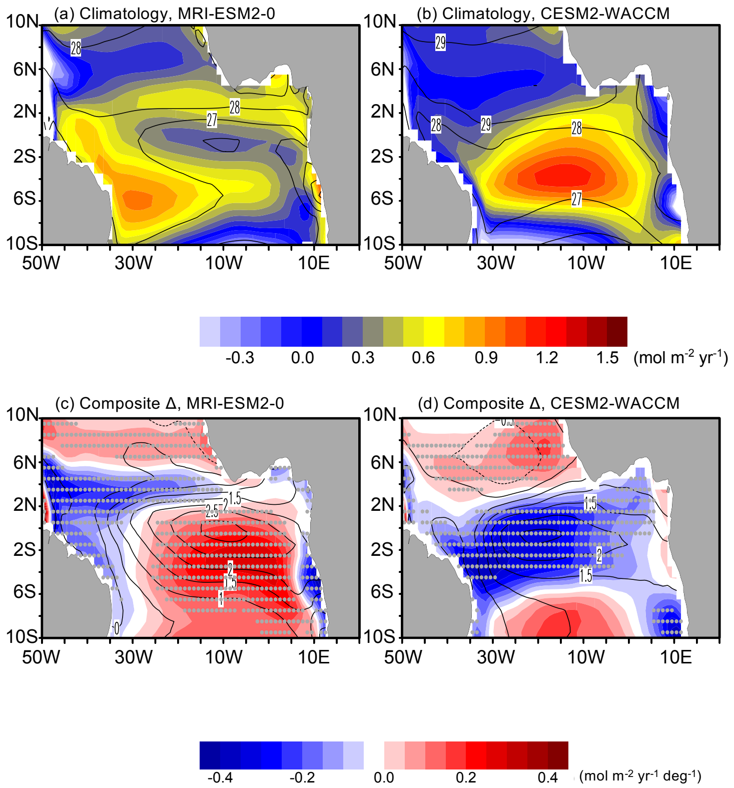

Figure S2. Simuated sea-air CO_2 flux and sea surface temperature (SST) from CMIP6 subset models.

Same as Fig.1c,d, but for the results of NorESM2, MRI-ESM2-0 (one of the best models) and CESM2-WACCM (one of the poor models) simulations. Composite anomalies are scaled by Atlantic-3 index composite anomaly between Niño and Niña events.

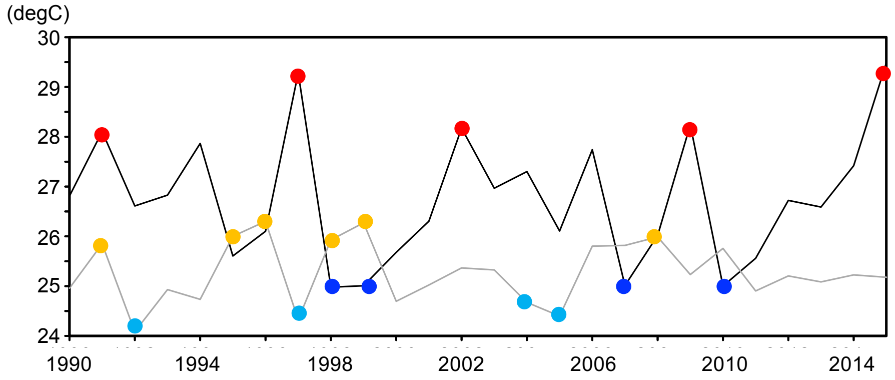

Figure S3. Time series of the observed sea surface temperature (SST) averaged 5S-5N/190E-240E (NINO3.4 box) and 3S-3N/340E-360E (Atlantic-3 box).
Red and blue dots are events of the Pacific Niño and Niña and orange and light blues are for the Atlantic Niño and Niña defined in this study, respectively.

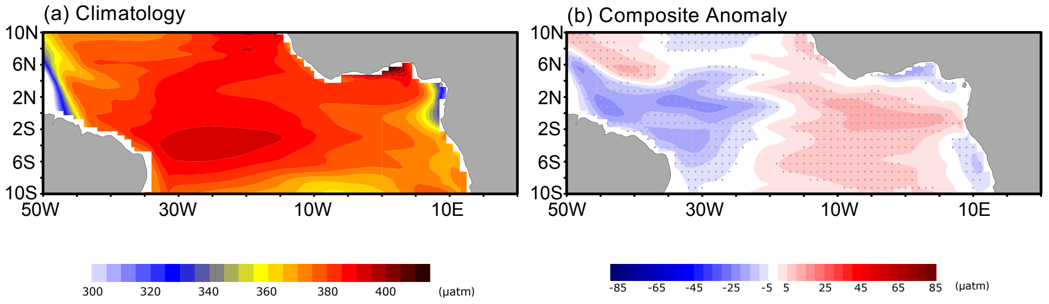

Figure S4. NorESM2 simulated *p*CO_2.
NorESM-prognostic *p*CO_2 cimatology and composite difference in *p*CO_2 between Atlantic Niño and Niña

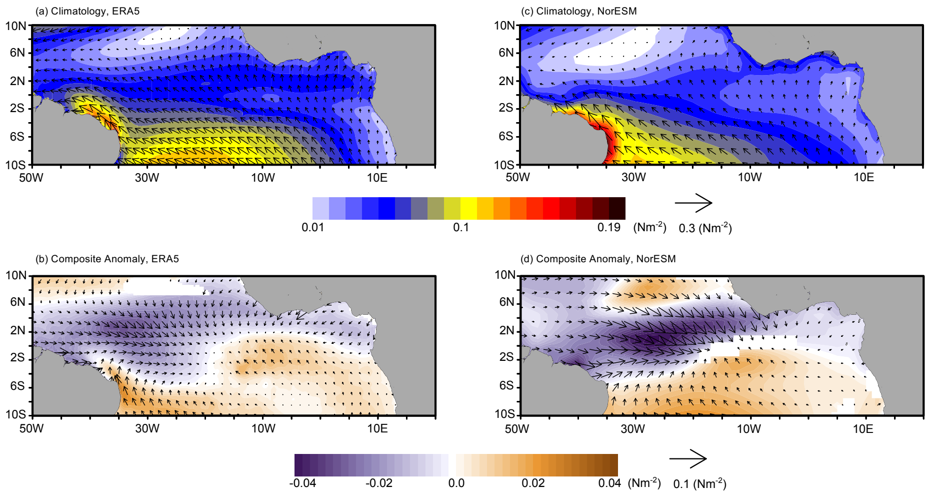

Figure S5. Observed and simulated wind stress.

Spatial patterns of (left column) observed and (right-column) simulated surface wind stress from the ERA5 reanalysis and NorESM2 model, respectively. The top row depicts long-term mean while the bottom row anomaly values during Atlantic Niño.

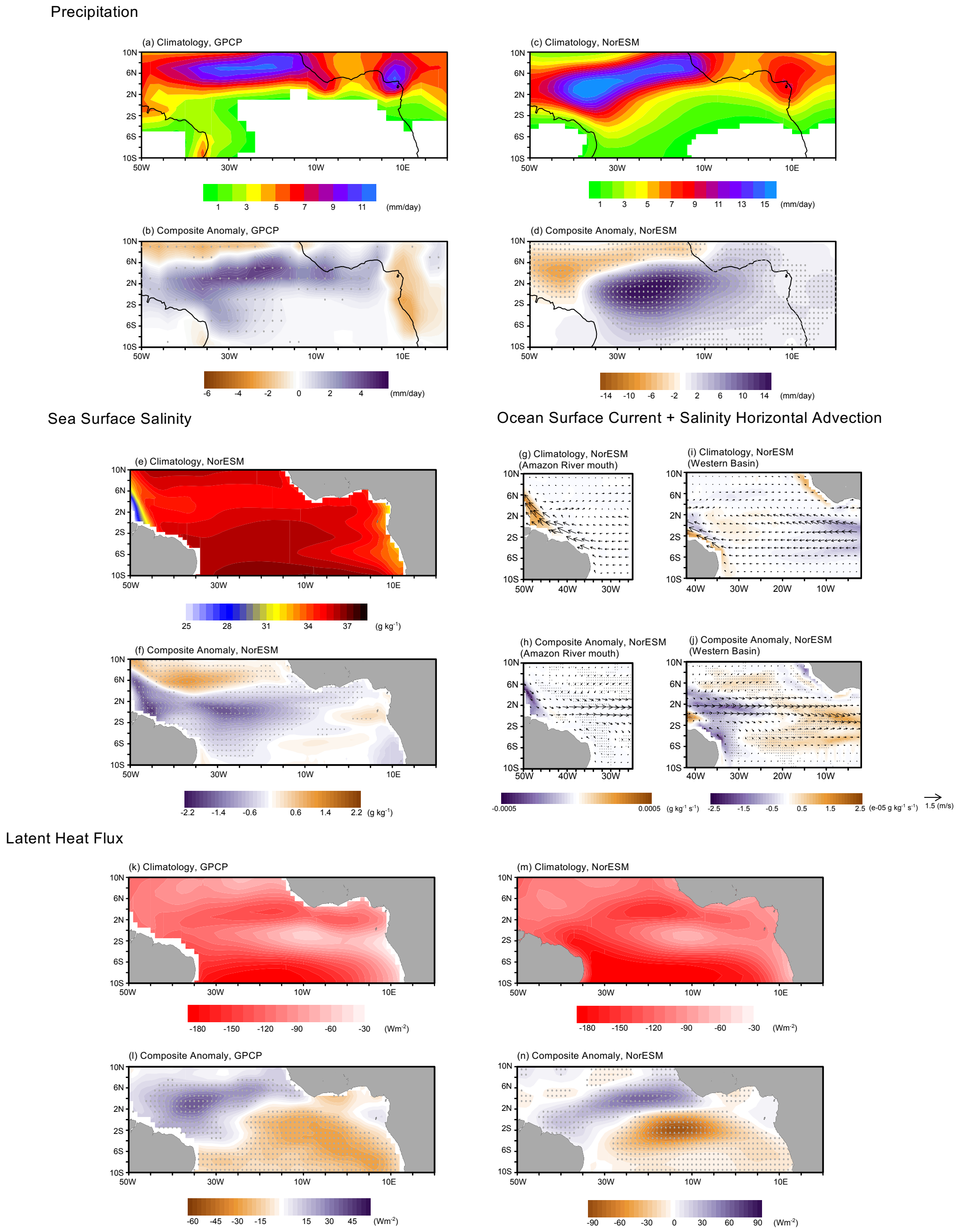

Figure S6. Precipitation, surface salinity, and latent heat flux.
Observed and NorESM2-simulated climatology and composite anomalies of variables relevant to freshwater. Same as Fig. S5, but for (top) the precipitation and (bottom) latent heat flux for (left) observation and (right) NorESM simulation. The middle panels are (left) sea surface salinity and (right) ocean surface current and horizontal salinity advection (two regions are shown : Amazon River Mouth and western basin due to the different value range) from the NorESM2 simulation. Note that the horizontal sainity advection is calculated from monthly data of SSS and ocean current and therefore, a contribution from the transient component is lacking. Note that observational sea surface salinity and ocean current monthly data with fine temporal duration is not available from 1990 to 2015. For the latent heat flux, negative denotes upward (ocean losses the heat and fresh water). Purplish (orangish) color for the

anomalies indicates tendecy of desalinification (salinification) at the sea surface. Gray dots denote 90% of significance level and only vectors whose zonal or meridional component anomalies are significance (90%) are shown. The observation data are GPCP(7) and TropFlux(8) for precipitation and latent heat flux, respectively.

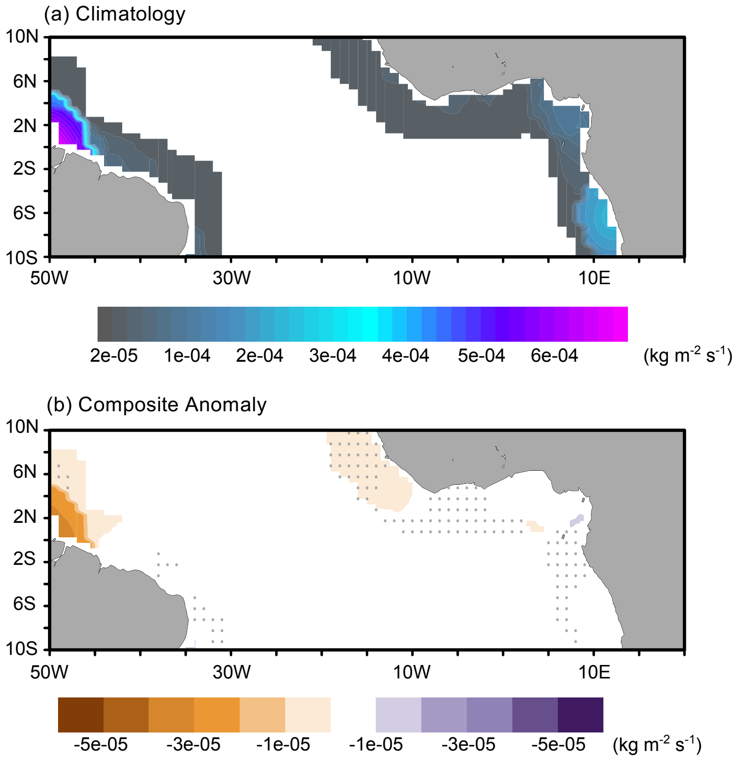

Figure S7. River runoff simulated by NorESM2.
Same as Fig.S5, but for the river runoff of the NorESM2.

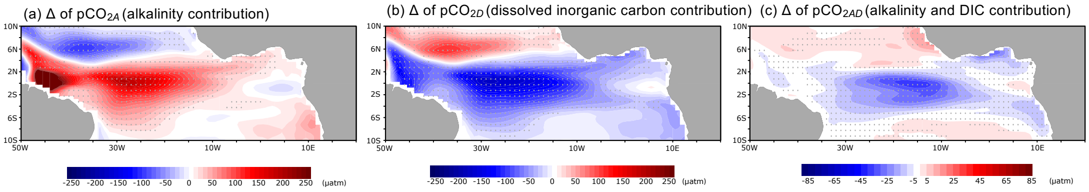

Figure S8. Dissolved inorganic carbon (DIC) and total alkalinity (ALK) contribution to *p*CO_2.
Same as Fig.3, but for pCO_{2A}, pCO_{2D}, and pCO_{2AD}

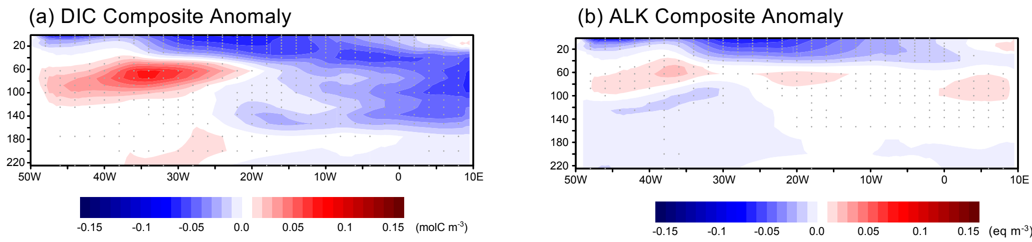

Figure S9. Vertical-longitude sections of dissolved inorganic carbon (DIC) and total alkalinity (ALK) anomalies simulated by NorESM2.
Same as Fig.2, but for the vertical-longitude section of (left) dissolved inorganic carbon and (right) total alkalinity averaged between 2S and 4N.

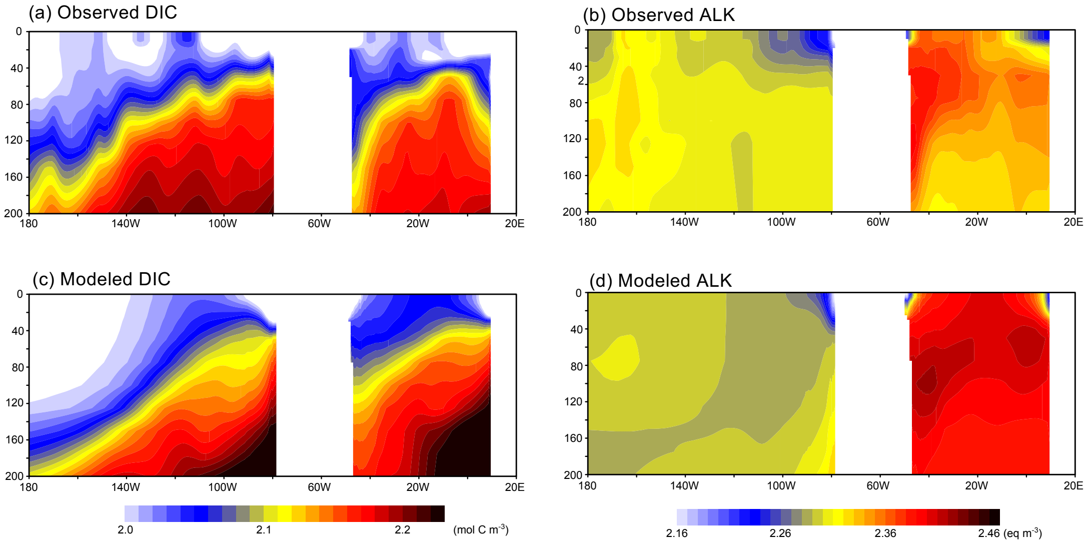

Figure S10. Vertical-longitude sections of observed and simulated dissolved inorganic carbon (DIC) and total alkalinity (ALK).
Depth-longitude section of annual-mean climatology of DIC and obtained from observation (top, GLODAP(9)) and NorESM simulation (bottom) averaged 3°S and 3°N.

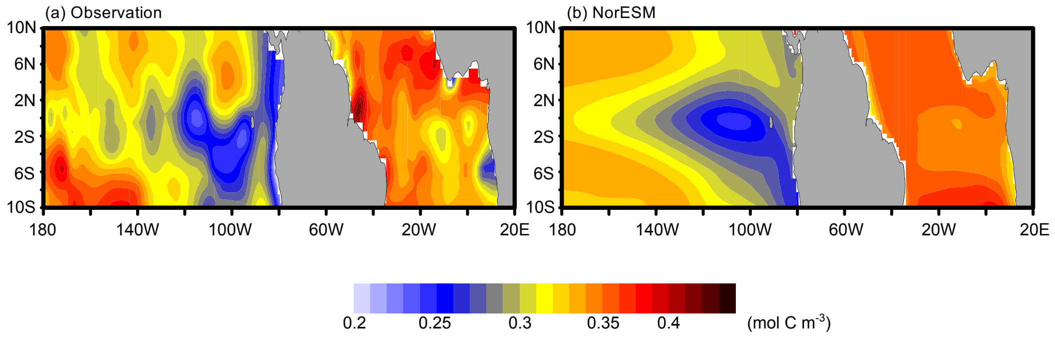

Figure S11. Observed and simulated carbonate ion.

Annual-mean climatology of carbonate ion (CO_3^{-2}) defined as total alkalinity (ALK) minus dissolved inorganic carbon (DIC) at the surface for (a) observation[9] and (b) NorESM2.

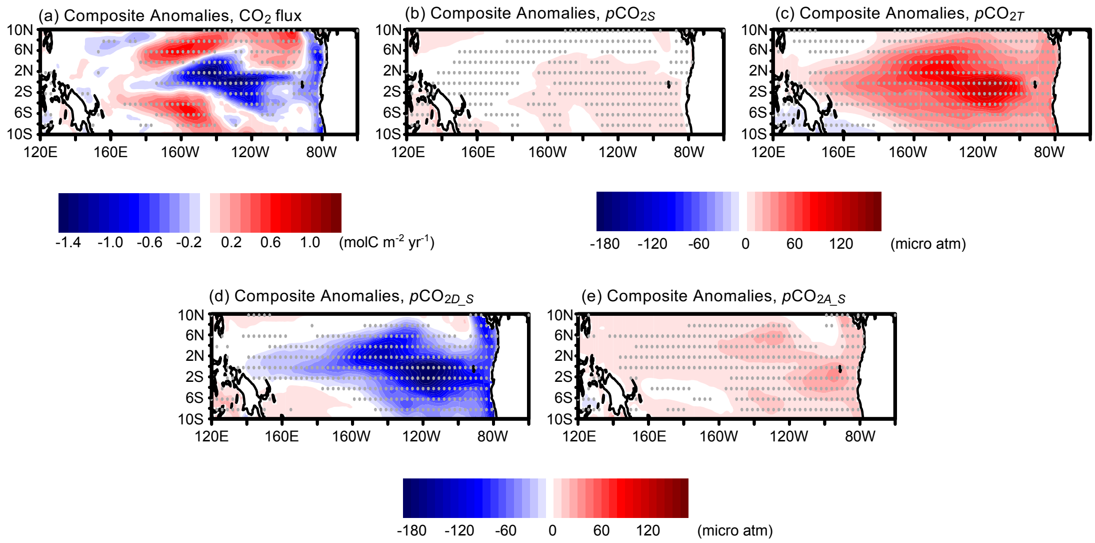

Figure S12. *p*CO_2 anomaly distribution in the case of Pacific El Niño simulated by NorESM2. Composite difference (defined as Δ)of (a) CO_2 flux, (b) pCO_{2S}, (c) pCO_{2T}, (d) pCO_{2D_S}, and (e) pCO_{2A_S} between El Niño and La Niña in November and December simulated by NorESM.

Supplementary References

1. Eyring V, Bony S, Meehl GA, Senior CA, Stevens B, Stouffer RJ, et al. Overview of the Coupled Model Intercomparison Project Phase 6 (CMIP6) experimental design and organization. Geosci Model Dev. 2016;9(5):1937-58.
2. Lewis E, & Wallce, D. W. R., . Program Devloped for CO2 System Claculation. In: Carbon Dioxide Information Analysis Center ORNL, U. S. Department of Energy, editor. Oak Ridge, Tennessee.
3. Reynolds RW, Smith TM, Liu C, Chelton DB, Casey KS, Schlax MG. Daily high-resolution-blended analyses for sea surface temperature. J Climate. 2007;20(22):5473-96.
4. Landschutzer P, Gruber N, Bakker DCE. Decadal variations and trends of the global ocean carbon sink. Global Biogeochem Cy. 2016;30(10):1396-417.
5. Landschutzer P, Laruelle GG, Roobaert A, Regnier P. A uniform pCO(2) climatology combining open and coastal oceans. Earth Syst Sci Data. 2020;12(4):2537-53.
6. Hersbach H, Bell B, Berrisford P, Hirahara S, Horanyi A, Munoz-Sabater J, et al. The ERA5 global reanalysis. Q J Roy Meteor Soc. 2020;146(730):1999-2049.
7. Adler RF, Sapiano MRP, Huffman GJ, Wang JJ, Gu GJ, Bolvin D, et al. The Global Precipitation Climatology Project (GPCP) Monthly Analysis (New Version 2.3) and a Review of 2017 Global Precipitation. Atmosphere-Basel. 2018;9(4).

8. Kumar BP, Vialard J, Lengaigne M, Murty VSN, McPhaden MJ, Cronin MF, et al. TropFlux wind stresses over the tropical oceans: evaluation and comparison with other products. Clim Dynam. 2013;40(7-8):2049-71.
9. Lauvset SK, Key RM, Olsen A, van Heuven S, Velo A, Lin XH, et al. A new global interior ocean mapped climatology: the 1 degrees x 1 degrees GLODAP version 2. Earth Syst Sci Data. 2016;8(2):325-40.
